# Three-Dimensional Non-Homogeneous Microstructure Representation Using 2D Electron Backscatter Diffraction Data for Additive-Manufactured Hastelloy X

**DOI:** 10.3390/ma17235937

**Published:** 2024-12-04

**Authors:** Liene Zaikovska, Magnus Ekh, Mohit Gupta, Johan Moverare

**Affiliations:** 1 Department of Engineering Science, University West, SE-461 86 Trollhättan, Sweden; mohit.gupta@hv.se (M.G.); johan.moverare@liu.se (J.M.); 2 Department of Material and Computational Mechanics, Chalmers University of Technology, SE-412 06 Gothenburg, Sweden; magnus.ekh@chalmers.se; 3 Department of Management and Engineering, Linköping University, SE-581 83 Linköping, Sweden

**Keywords:** powder bed fusion–laser beam (PBF-LB), electron backscatter diffraction (EBSD), representative volume element (RVE), crystal elasticity finite element (CEFE), computational homogenization (CH)

## Abstract

Additive manufacturing (AM) methods like powder bed fusion–laser beam (PBF-LB) enable complex geometry production. However, understanding and predicting the microstructural properties of AM parts remain challenging due to the inherent non-homogeneity introduced during the manufacturing process. This study demonstrates a novel approach for 3D microstructure representation and virtual testing of non-homogeneous AM materials using 2d electron backscatter diffraction (EBSD) data. By employing the representative volume element (RVE) method, we reconstruct the 3D microstructure from 2D EBSD datasets, effectively capturing the grain morphological characteristics of PBF-LB-produced Hastelloy X. Using validated RVE data, we artificially generate combinations of two grain textures to gain deeper insight into locally affected areas, particularly the stress distribution within the interfaces, as well as global material behavior, exploring non-homogeneity. Computational homogenization (CH) utilizing a crystal elasticity finite element (CEFE) method is used to virtually test and predict directional elastic properties, offering insights into relationships between microstructure evolution and property correlation. The experimentally validated results show a strong correlation, with only 0.5–3.5% correlation error for the selected grain tessellation method. This consistency and reliability of the methodology provide high confidence for additional virtual tests predicting the properties of non-homogeneous, artificially generated combined-grain structures.

## 1. Introduction

Additive manufacturing (AM) has emerged as a transformative technology, enabling the creation of intricate geometries and highly customized materials with exceptional precision. Among various AM techniques, the powder bed fusion–laser beam (PBF-LB) process stands out for its ability to produce complex parts with excellent mechanical properties [1]. This process has been widely adopted in industries such as aerospace, automotive, and medical [2,3,4], especially for manufacturing high-performance materials such as the Hastelloy X superalloy, which is also utilized in this study. Hastelloy X is a nickel-based superalloy known for its exceptional strength, oxidation resistance, and ability to withstand high temperatures, making it an ideal candidate for demanding applications [5]. Understanding the microstructural properties of this material, as well as other AM materials is crucial for optimizing their performance and reliability. However, this task is challenging due to the inherent non-homogeneity introduced during manufacturing. To better understand material behavior, the electron backscatter diffraction (EBSD) technique can be used to gain insights into the microstructural characteristics of materials, including grain orientation, phase distribution, and crystallographic texture [6,7]. EBSD provides high-resolution information about the orientation of individual grains within polycrystalline materials, allowing deeper knowledge of the relationships between microstructure and mechanical properties. By mapping the crystallographic structure of materials, EBSD helps in identifying grain boundaries, also enabling analyzing material behavior during the build process, such as recrystallization, phase transformations, and texture evolution, which are crucial for predicting how materials respond under different loading conditions [8]. As an example, the study by Jie Sun et al. [9] demonstrates the deformation behavior of magnesium alloys, specifically how microstructural characteristics, such as grain boundary misorientation, affect mechanical properties like ductility and formability. The study investigated the effects of different slip systems and twinning mechanisms during tensile deformation. By utilizing the EBSD technique and a misorientation distribution function (MDF) analysis, the role of grain boundary compatibility and misorientation in enhancing the deformation capabilities were examined.

It is also acknowledged that alternative techniques, such as X-ray tomography [10], can be used to analyze microstructures. In a study by Veerappan Prithivirajan et al. [11], high-energy X-ray diffraction and tomography experiments are conducted to validate a crystal plasticity finite element (CPFE) model, focusing on microstructure-sensitive crack initiation locations. A comprehensive understanding of the grain behavior of AM polycrystalline microstructures is clearly necessary. A study by Amir Mostafaei et al. [12] delves deeper into the complex interactions in metal AM, focusing on the dynamics of keyhole mode, the role of numerical modeling, and a comprehensive classification of defects. The study emphasizes the importance of understanding these aspects to optimize AM processes, enhance part quality, and minimize defect formation. It has been shown that defects contribute significantly to non-homogeneity and are frequently encountered during the AM material build process. This issue is still relatively new in research and requires further understanding, particularly in how it affects the final material performance. In a previous study where directional elastic properties and texture-breaking effects are investigated [13], it was shown that non-homogeneity is a contributing factor to the variation in the properties of powder bed fusion–electron beam (PBF-EB)-manufactured material. However, the investigation was limited by the challenge of representing the texture-breaking effects requiring extensively large grain data. This limitation restricted exploration into how this phenomenon affects material on a local-scale level. Furthermore, the study did not examine the local interfaces of mixed-grain structures, focusing exclusively on the property prediction at a macroscale level.

In our study, we address the latter-mentioned limitations by investigating isotropic textured-grain structures produced via the powder bed fusion–laser beam (PBF-LB) process with a methodology applicable to large EBSD datasets, as well as a variety of microstructures, enhancing the understanding of directional elastic properties and stiffness not only in bulk materials but also when implementing non-homogeneous combined-grain structures. In this study, we utilize EBSD microstructure information as input to generate the 3D representative volume element (RVE) models and to predict the directional elastic properties. EBSD data have frequently been utilized as input for micromechanical simulations, as demonstrated in studies such as [14,15,16,17,18]; however, most studies use virtual testing, implementing synthetic RVE models, and primarily examine bulk material behavior [19], assuming no local variations within the material. The most common method for 3D microstructure representation is to use 3D EBSD serial sectioning data [20]. Acquiring 3D EBSD is costly and time-consuming, as standard microscopic procedures can only produce 2D surface maps. In our contribution, we present a methodology that implements a 3D RVE representation using only one single 2D EBSD section. By incorporating this approach, we achieve a close approximation to experimental data and demonstrate an optimal method for efficiently representing both bulk material and introduced non-homogeneity, named as combined structure. In many cases, these types of microstructures are challenging to produce and thus are not yet available for validation. However, as this study demonstrates, the simulation can be implemented with minimal effort, making it valuable as a preliminary step before physical production.

Additionally, we observe that PBF-LB-processed Hastelloy X exhibits different texture evolutions depending on the thickness of the produced material samples. Materials thicker than 1 mm predominantly display a combination of <001>, <011>, and <111> crystallographic orientation perpendicular and parallel to build direction (BD), while 1 mm thin samples develop grain structures with a strong <001> crystallographic orientation in the perpendicular plane to BD while remaining at the same <011> orientation parallel to the BD.

Understanding these variations is crucial for predicting material performance, as the different grain structures present in PBF-LB-processed materials significantly affect their mechanical properties. It is important to note that Ni-based superalloys are particularly challenging due to their sensitivity to various types of crack formation, which often results in processing difficulties and metallurgical defects, as the authors of the study in [21] describe. These issues arise from the complex microstructure and high-strength characteristics of the material, which can lead to brittleness and reduced ductility. As a result, there are often processing problems such as hot cracking, strain-age cracking, and fatigue failures, which complicate manufacturing and limit the material performance. Defects such as void distributions are significant for microstructure analysis in AM alloys. However, given that the directional elastic properties in our study closely matched experimental data, a void distribution analysis was excluded based on the assumption of minimal or negligible porosity in the tested material.

Our contribution focuses on exploring methodologies for AM material representation, modeling, and virtual testing to gain deeper insights into the grain structure transition regions where crack propagation commonly initiates [22]. The presence of non-homogeneity makes it challenging to study and measure these properties experimentally. However, accurately measuring directional stiffness when introducing non-homogeneity is essential. Virtual testing has emerged as a highly effective approach for generating extensive datasets exploring the tendencies of this specific material behavior. Implementing the crystal elasticity finite element (CEFE) using a computational homogenization approach is particularly useful for studying such structures. The interfaces, where different microstructures meet, play a crucial role in determining the local stress behavior which directly impacts the overall mechanical performance of the material. Despite its importance, this phenomenon has been the subject of very few studies [23,24], and even fewer have focused on predicting the directional properties of this type of microstructure evolution. The RVE method allows for the analysis of these critical regions, providing valuable insights into how local effects and non-homogeneities impact the macroscopic properties of the material. This insight is particularly useful not only for understanding the local behavior of the mixed-grain structures but also in scenarios like tailoring materials and microstructure [25,26] by implementing hybrid AM with mixed materials or processes [27], as well as when introducing component repair [28,29]. Understanding these material types is essential for designing AM components that meet critical performance standards, leading to more reliable and efficient applications of AM technology across diverse industries.

By validating virtually tested microstructure, we predict the directional elastic properties of PBF-LB-manufactured bulk Hastelloy X material with a precision of 0.5–3.5% correlation error, confirming the reliability of our methodology. This approach is then extended to study artificially generated combined-grain structures considering non-homogeneity. It can be shown that the applied methodologies are reliable and offer numerous possibilities for modeling such microstructures using real EBSD material data, thereby expanding knowledge in the AM field. These approaches do not require costly equipment nor time-consuming simulations, making them suitable for industrial environments for exploring local and global AM material behavior of distinct combinations.

## 2. Materials and Methods

### 2.1. Hastelloy X

Hastelloy X is a nickel-based alloy composed primarily of nickel, chromium, iron, and molybdenum, recognized for its exceptional oxidation resistance and high-temperature strength [5]. Its FCC (face-centered cubic) crystal structure contributes to its superior mechanical performance and resistance to oxidation at elevated temperatures. Hastelloy X can withstand temperatures up to 1200 °C [30], and its primary phase is the γ phase which does not heavily rely on secondary phase precipitates enhancing its formability and weldability. The elastic constants C11 = 227.7 (GPa), C12 = 155.5 (GPa), and C44 = 118.7 (GPa) are taken from the literature and used as inputs for the simulations in this study. Single-crystal experimental tests are seldom performed; however, the selected elastic constant values were validated in prior studies [31,32], demonstrating a close correlation to observed results. This validation supports their use in our study as well. The chemical composition of Hastelloy X is listed in Table 1.

### 2.2. PBF-LB Process Sample Preparation

**Figure 1:**
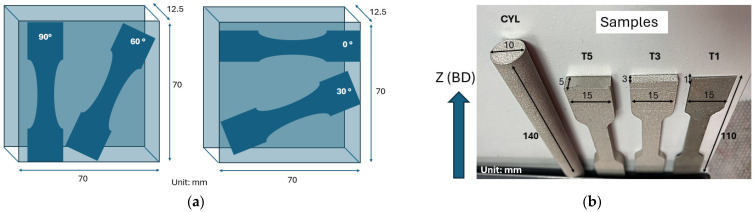
(**a**) Schematic representation of the samples used for cyclic elasticity testing; (**b**) samples used for EBSD data generation where the normal to build direction (BD) at the mid-section of the specimens are selected. Notation ‘CYL’ corresponds to cylinder, and ‘T5’, ‘T3’, and ‘T1’ correspond to bars with thicknesses of 5, 3, and 1 mm.

In this study, multiple samples are produced using the PBF-LB process. The samples shown in Figure 1a were machined from the hatch region of a block geometry to conform to the required test sample shape, which was subsequently used in the experimental tests. Here, a schematic representation of the machined samples is provided. Figure 1b illustrates samples with varying geometries, which were used for EBSD data collection and subsequently served as input for the RVE models investigated in 0°, 90°, 60°, 45°, 40°, and 30° angle directions. To ensure accuracy in EBSD analysis and grain tessellation, all samples were meticulously polished, as their sensitivity requires minimizing surface scratches that could interfere with analysis. All sample geometries are manufactured using 20 µm layer thickness.

The powder used in this process follows Siemens standards and is applied using a customized EOS M290 3D printer (https://uk.eos.info/en-gb, accessed on 17 September 2024). Each geometry is designed to assess the structural and mechanical characteristics, allowing for a comprehensive analysis of how geometry and dimensional variation influence material characteristics as well as directional elastic properties.

### 2.3. Experimental Procedure

#### 2.3.1. Cyclic Elasticity Test

**Figure 2:**
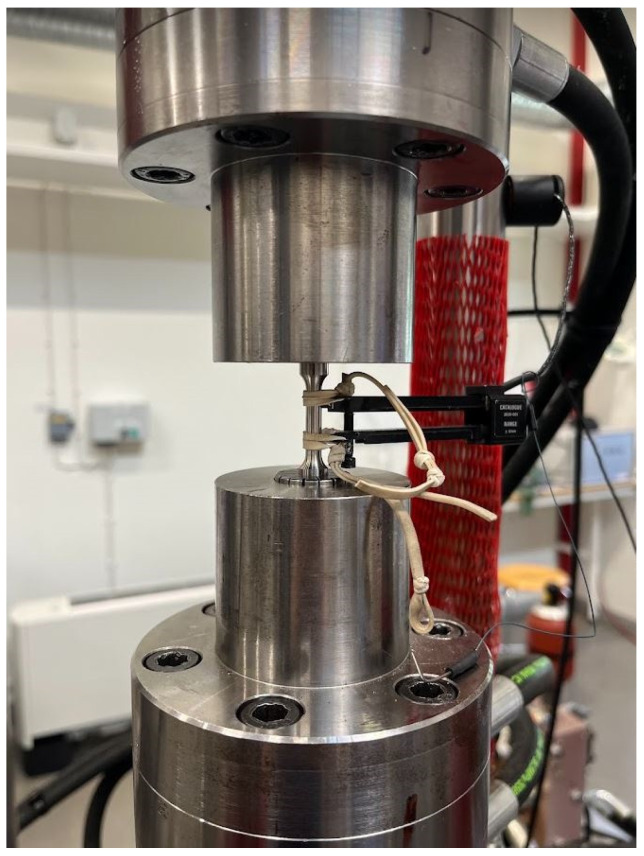
Cyclic elasticity test equipment.

Two types of tests—cyclic elasticity and tensile tests—were performed to determine the elastic modulus and to generate the tensile stress–strain curve. Findings from a previous study [13] and this study indicated that cyclic elasticity tests yield more accurate results for determining Young’s modulus than tensile tests. Therefore, this method was also used in the current work. Cyclic experiments are frequently used to evaluate material failure [33] or fatigue life [34]. Moreover, they can be utilized to conduct cyclic elasticity testing [35], which accurately measures and identifies the elastic modulus while detecting potential changes in mechanical properties induced by cyclic loading. To perform cyclic elasticity tests, a specialized cyclic loading machine, equipped with load and displacement sensors, has been utilized, as illustrated in Figure 2.

This machine is capable of applying precise, controlled cyclic loads to test specimens machined according to the standardized dimensions specified in ASTM E8/E8M-24 [36]. In this study, the specimens include dog-bone-shaped tensile samples, carefully prepared with smooth, defect-free surfaces to minimize stress concentrations and ensure accurate results. Cyclic elasticity tests were conducted using a servo-hydraulic fatigue test rig equipped with an Instron ±50 kN load cell, a 12.5 mm gauge length extensometer, and an Instron 8800 control system. The samples were subjected to 10 load cycles within a stress range of ±100 MPa, and the elastic modulus was determined as the average slope of the stress–strain curve within this range.

#### 2.3.2. EBSD Data Generation

**Figure 3:**
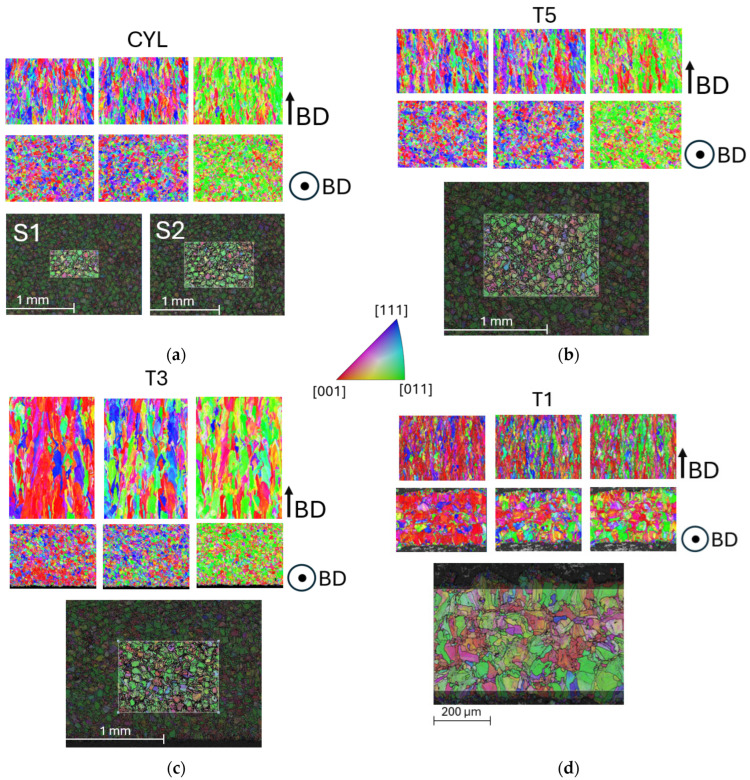
Chosen EBSD of normal to BD sections for (**a**) CYL, (**b**) T5, (**c**) T3, and (**d**) T1 samples. Notation ‘S1’ and ‘S2’ refers to EBSD datasets 1 and 2. Coloring indicates inverse pole figure (IPF) maps parallel and normal to BD, respectively.

From an industrial perspective, selecting the appropriate sections for EBSD generation and analysis is critical to obtain accurate and representative data. In this study, the EBSD sections were consistently chosen from the center of the observed area, as the grain morphology in this region is most likely representative of the bulk of the sample material studied. The goal was to maximize the amount of grain data collected by focusing on regions that provided the most detailed and comprehensive information about the material microstructure. Specifically, sections were selected at the center of the sample surface, with the section plane oriented perpendicular to the build direction seen in Figure 3. This orientation is optimal for capturing grain structure and texture which serves as input for the tessellation of RVE models.

The samples designated for EBSD were mounted in Bakelite and subjected to mechanical grinding from 500 to 4000 Grit. Polishing was performed with diamond suspensions ranging from 3 to 14 µm, finishing with OP-U colloidal silica suspension. EBSD mappings were conducted on a Zeiss Gemini 450 SEM (Zeiss, Oberkochen, Germany) equipped with an Oxford Instruments EBSD system (Oxford Instruments, Abingdon, UK), operating at 15 kV. Choosing sections with this orientation ensures that critical features such as grain size, position, and crystallographic orientation are effectively captured. A detailed explanation of the grain representation methodology is provided in the following Section, and the grain data characterization settings are implemented using the MTEX toolbox, MATLAB 24.1.0 (R2024a) [37] and are outlined in Table 2. This pixel data represent grain information, initially imported as raw EBSD microstructure data.

The resolution information provides an indication of the size of the EBSD section. In this study, the balance between section size and the number of grains has been a critical factor which has also been a limitation. In some cases, denoising was necessary due to the scattering of small grains, which is commonly observed in PBF-LB microstructures. When denoising is applied, it may allow for smoother grain boundary transitions and more accurate grain orientation definitions. Table 2 also shows that a higher threshold angle value can be applied when a smaller step size is used during EBSD data acquisition, which is a preferable outcome. The threshold angles are selected based on the overall microstructure and individual grain quality, taking into account subsequent process steps where the grain count must also be considered. This step may be iterative, as the grain tessellation process may reveal potential issues that require adjustments to the threshold angles. The standard mean values of aspect ratio are collected from EBSD data and applied across all sample cases to represent realistic grain morphology, even though a previous study [38] has shown that for predicting elastic properties, the grain aspect ratio is not a critical parameter in microstructure representation.

### 2.4. Grain Representation

**Figure 4:**
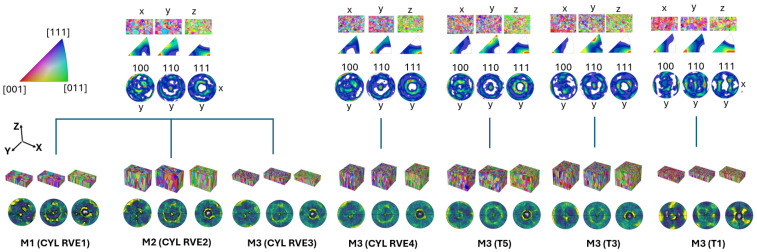
RVE representation and the corresponding EBSD sections including pole figure (PF) and inverse pole figure (IPF) maps.

This Section explores various methods for grain representation, as the RVE model setup is detailed in Table 3. Tessellation methods M1–M3 are implemented to evaluate their impact on the input data and their tessellation capabilities. Tessellation methods implemented in RVE sample cases are presented in Figure 4, and a detailed description is presented in upcoming sub-sections. A combined method (CM) is used to generate combined structures and investigate the effect of non-homogeneity and is presented with a detailed description in Section 2.4.4.

The primary focus of this study is to develop a methodology that closely replicates the actual grain morphology while also considering the capabilities of using NEPER [39] for grain tessellation and RVE model creation. Algorithms in NEPER are an iterative, optimization-based approach that combines tessellation, morphology, and orientation assignment to create realistic 3D polycrystalline structures. In this study, Voronoi-based tessellation approach [40] and specific Euler–Bunge mean orientation angles are implemented in all RVE cases. A Voronoi diagram is constructed by partitioning the three-dimensional space into cells based on a given set of seed points $P=\left\{p(1),\ldots ,\;p(n)\right\}$. Each cell is associated with a seed point p(i), such that every point within the cell is closer to p(i) than to any other seed point p(j), where j≠i, according to a chosen Euclidean metric. Initially, the four RVE tessellation methods are compared using the CYL sample grain data from EBSD S1 and S2, as illustrated in Figure 3a. Each of the implemented methods is also described in the following Subsections. The most effective RVE method is then chosen to be implemented across all sample grain tessellations. Additionally, combined structures are artificially generated in a distinct manner to facilitate the integration of real EBSD data, as well as various RVE thicknesses being used to examine their effect on property prediction. The impact of RVE thickness in the Z-direction was initially studied using a CYL sample to ensure it does not influence the results. The thickness of the RVE sample, T1, was then reduced to fit within one of the combined structures, where T1 is integrated with the CYL sample structure.

The RVE sizes vary based on structural complexity and the availability of EBSD data where the number of grains determines the size of an RVE. In the case of the T1 sample, due to limited material availability, most of the hatch area is selected, though it contains fewer grains compared to other samples. For samples with extensive data, considerations must still be made for tessellation capabilities, meshing feasibility, and computational storage. Applying the presented methodology requires a careful balance between grain information, tessellation capabilities, and computational constraints.

#### 2.4.1. Method 1 (M1)

The RVE1 model is tessellated using M1 and CYL S1 sample, where the real grain morphology is captured by incorporating as much actual input data as possible to achieve an accurate and representative result, as shown in Figure 4. M1 involves implementation of actual grain sizes, centroid coordinates, mean grain orientations, and grain aspect ratios which makes it very difficult for NEPER to optimize the given input through the iterative process using following code:

neper -T -n (grain count) -id 1 -morphooptiini ‘coo:file(coos)’ -morpho ‘size:file(cell_size),aspratio(x,y,z)’ -ori ‘file(mean_ori,des=euler-bunge)’ -statcell size -statseed x,y,z -domain ‘(RVE size)’ -o RVE1

The tessellation process is time-consuming, and since only one data plane is used, the software struggled to achieve the correct tessellation while considering all the input data simultaneously. This also made the meshing of the RVE models challenging. In general, this method often resulted in either failed tessellation or meshing, leading to an unstable and unreliable process. However, it is important to note that using multiple EBSD data planes would significantly enhance the stability of this method and provide highly accurate representation of the microstructure [41].

#### 2.4.2. Method 2 (M2)

The RVE2 grain model is tessellated using M2, with CYL S1 sample grain data collected from the EBSD, as illustrated in Figure 4. Similar to the RVE1 case, the goal is to preserve as much actual grain morphology as possible. However, to aid NEPER during the tessellation process, centroid coordinates were excluded in this case implementing following code:

neper -T -n (grain count) -id 1 -morpho ‘size:file(cell_size),aspratio(x,y,z)’ -ori ‘file(mean_ori,des=euler-bunge)’ -statcell size -statseed x,y,z -domain ‘(RVE size)’ -o RVE2

This adjustment allowed for NEPER to determine the most optimal grain positions, resulting in a faster and more efficient tessellation process. Additionally, the generated grain shapes feature smoother surfaces, which contributes to a more stable meshing process. It is evident that by using the input of 2D grain morphology and M2 tessellation method, the real microstructure orientation can be accurately replicated, even though the grains do not retain their original positions. When comparing the NEPER PF with the EBSD PF maps, the texture information remains nearly identical.

#### 2.4.3. Method 3 (M3)

The M3 tessellation method uses even less input data compared to M1 and M2 implementing following code:

neper -T -n (grain count) -id 1 -morpho ‘aspratio(x,y,z)’ -ori ‘file(mean_ori,des=euler-bunge)’ -statcell size -statseed x,y,z -domain ‘(RVE size)’ -o RVE3/RVE4

In this approach, only grain orientation and aspect ratios are specified as input parameters to represent the sample of CYL S1 and S2, as shown in Figure 4. With M3, NEPER is able to reorganize grain positions and adjust grain sizes, making the tessellation process both faster and more efficient. By reducing the number of input parameters to only grain orientation and aspect ratio, NEPER has greater flexibility to optimize grain distribution and sizes during the tessellation. This streamlines the process, significantly reducing the complexity while still providing a reasonable representation of the microstructure. The EBSD S1 is compared with S2 where a larger EBSD area with increased grain count and a larger RVE size has been implemented to increase the representation accuracy. Due to the stability of M3 in the tessellation process, it was applied to all other samples—T5, T3, and T1.

It can be noticed that the grain orientation of T5 sample closely resembles that of the CYL sample. In both cases, the crystallographic directions are aligned in a distinct pattern: <001> is predominant in the plane parallel to the X-direction, <111> dominates the plane parallel to the Y-direction, and <011> is dominant in the plane parallel to the Z-direction. This specific combination of orientations creates a distinctive material texture that might significantly impact on the material properties, suggesting behavior with isotropic characteristics. When examining the T3 sample and its microstructure evolution, changes in texture begin to appear within the crystallographic planes parallel to the X- and Y-directions. However, the orientation in the Z-direction remains consistent with that of the CYL and T5 samples. An important observation is that the thickness of the sample has begun to influence its microstructural characteristics. In thinner structures, a distinct texture starts to emerge, leading to a noticeable shift in crystallographic orientation. This change results in the development of slight anisotropy, indicating that thickness plays a critical role in the evolution of grain orientation and overall texture. As the structure becomes thinner, the texture in the crystallographic planes along the X- and Y-directions begins to exhibit a pronounced <001> orientation. This effect is particularly seen in the T1 sample shown in Figure 4.

Given the clear impact of geometric aspects on material behavior, it becomes crucial to examine how these textures interact with non-homogeneous material properties. To address this, combined microstructures are artificially generated, and the methodology is detailed in the upcoming subsection. The focus is on understanding their specific effects when later applied in virtual testing.

#### 2.4.4. Combined Method (CM)

**Figure 5:**
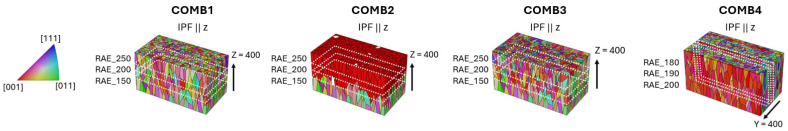
Studied microstructure of combined cases and representative area element (RAE) sections. Notation ‘COMB’ refers to combined cases.

The CM uses the M3 setup with a difference in input grain orientation data, which is assembled in a separate file of Euler–Bunge angles. For the artificially generated combined cases, various interface sections, referred to as representative area elements (RAEs), are examined as shown in Figure 5. The orientation of the interface plane is normal to the BD and position—at the center of the RVEs, as well as plane offsets above and below the middle plane. In the case where segmented texture is combined with random grain orientation, the plane parallel to the BD is studied instead, due to the orientation of the grain interfaces of this specific structure.

**Figure 6:**
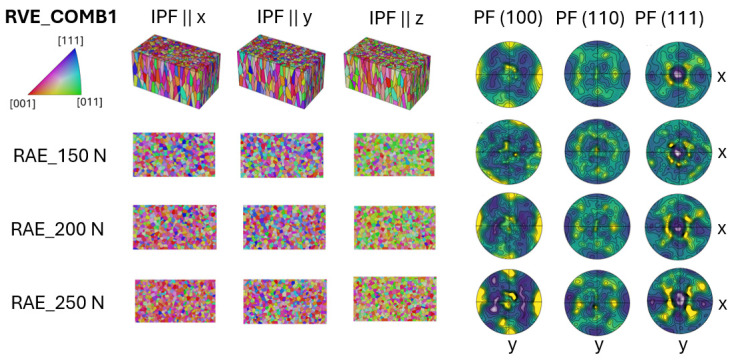
Three-dimensional RVE representation of the COMB1 case using combined method (CM) and corresponding 2D interfaces including pole figure (PF) maps. Notation ‘N’ indicates plane Normal to build direction (BD).

**Figure 7:**
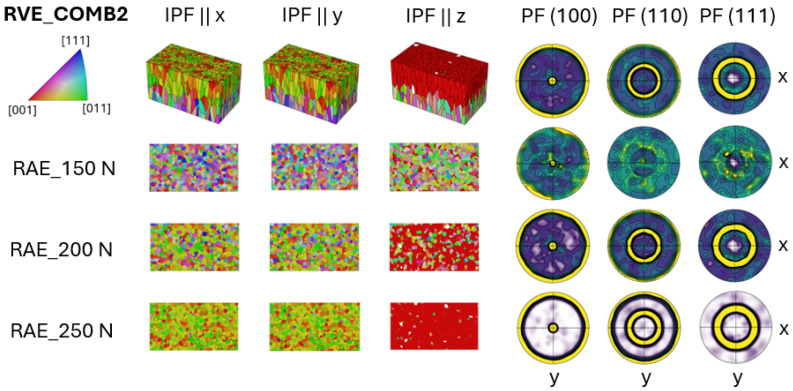
Three-dimensional RVE representation of the COMB2 case using combined method (CM) and corresponding 2D interfaces including pole figure (PF) maps. Notation ‘N’ indicates plane normal to build direction (BD).

**Figure 8:**
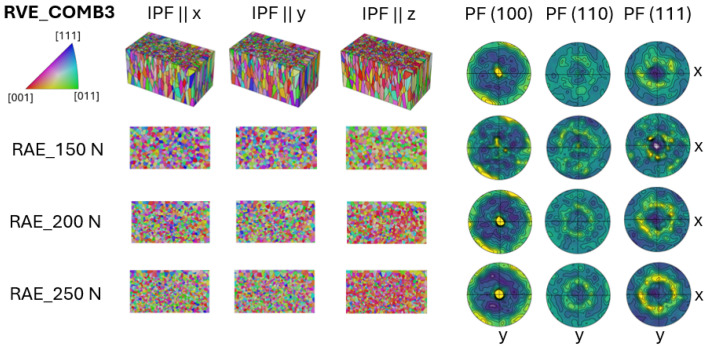
Three-dimensional RVE representation of the COMB3 case using combined method (CM) and corresponding 2D interfaces including pole figure (PF) maps. Notation ‘N’ indicates plane normal to build direction (BD).

**Figure 9:**
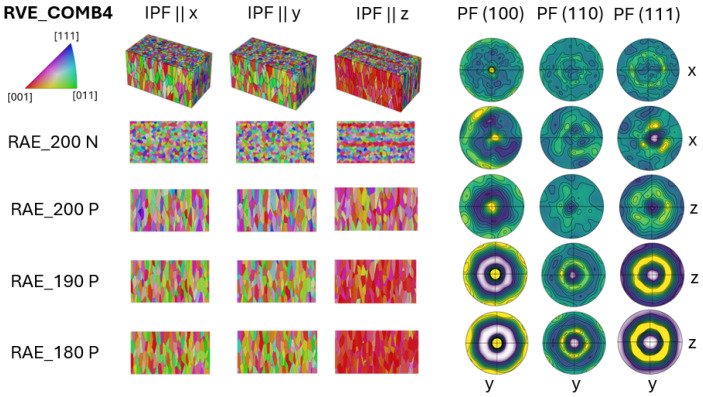
Three-dimensional RVE representation of the COMB4 case using combined method (CM) and corresponding 2D interfaces including pole figure (PF) maps. Notation ‘N’ indicates plane normal to build direction (BD), and ‘P’ indicates plane parallel to build direction (BD).

The CM is used to create RVE models featuring texture combinations that represent transition regions between different grain structures. For the cases COMB1, COMB2, and COMB3, this is achieved by artificially assembling 2D EBSD grain data to reflect the transitional textures in 3D, as illustrated in Figure 6, Figure 7 and Figure 8. In the COMB4 case, grain orientation data are generated based on the grain centroid coordinates. When this is used as orientation input, it results in the segmented texture shown in Figure 9.

These RVE models are also analyzed by extracting interface texture information from 2D RAE sections at various positions. Four different cases are tessellated, where the main difference is the implemented textured grain orientation. In the COMB1 case seen in Figure 6, the EBSD data from CYL and T1 samples are combined to represent the intersection of these two materials.

The transition between CYL and T1 textures is clearly observed in the RAE sections shown in Figure 6. Both the PF and IPF maps reveal a less pronounced <001> grain orientation in the CYL section, with the intensity increasing as passing the interface transition toward the T1 structure part.

In the COMB2 case, shown in Figure 7, the combination of CYL texture and an ideal <001> grain orientation makes the contrasting texture differences even more apparent. The interface section exhibits a dominant <001> crystallographic orientation, which becomes increasingly pronounced as it moves through the transition. Despite this strong orientation, there are still some residual regions where the CYL texture persists. These areas of CYL texture are less prominent but remain noticeable, indicating that the transition between textures is gradual rather than abrupt.

In the COMB3 case, the combination of the CYL sample and the PBF-EB-processed microstructure, collected from the previous study [13], is tessellated, and the representation shown in Figure 8.

It is important to note that the PBF-EB part was created using the same tessellation method as the CYL part. This method utilizes built-in functions in NEPER to distribute grain sizes and positions while only requiring input of grain orientations and aspect ratios. This aspect leads also to the limitation of defining a different aspect ratio value for the PBF-EB part that would better represent the distinct grain morphology typical of PBF-EB grains. This limitation arises for two reasons: First, the method used for the combined cases is based on a simpler approach where the grain size and orientation distributions are not provided as exact inputs. Second, the built-in functions in NEPER have limited capability in customizing the aspect ratio values in the same RVE. Despite this, the transition region can still be observed, with the texture gradually developing.

The COMB4 case represents a unique transition region compared to the other combined cases, primarily due to the interaction between two distinct grain orientation structures: a strong <001> texture and random grain orientations, as shown in Figure 9.

This interaction creates a complex microstructure, where several segmented parts with distinct grain characteristics form during the transition. The strong <001> texture dominates one part of the material, which leads to enhanced stiffness along BD but reduced properties in other directions. On the other side of the transition, random grain orientations are introduced. These grains lack any preferred alignment and exhibit more isotropic properties, meaning their mechanical response is relatively uniform in all directions. This randomness in orientation contrasts sharply with the <001> texture, creating interfaces where these two structures meet. The transition region between the strong <001> texture and random grain orientations is complex, causing localized variations in grain boundary energy and density. This can significantly influence material behavior, especially in terms of crack propagation, deformation, and mechanical strength. The mixing of these two textures results in a gradual shift in the grain structure across the transition region, rather than a sharp boundary. The strong <001> texture slowly dissipates, blending into the random grain orientation, creating a gradient-like texture. The characteristics of this transition can affect how the material responds to stress, heat treatment, or other processes, making this an important area of study for applications that require tailored material properties.

### 2.5. Computational Homogenization

**Figure 10:**
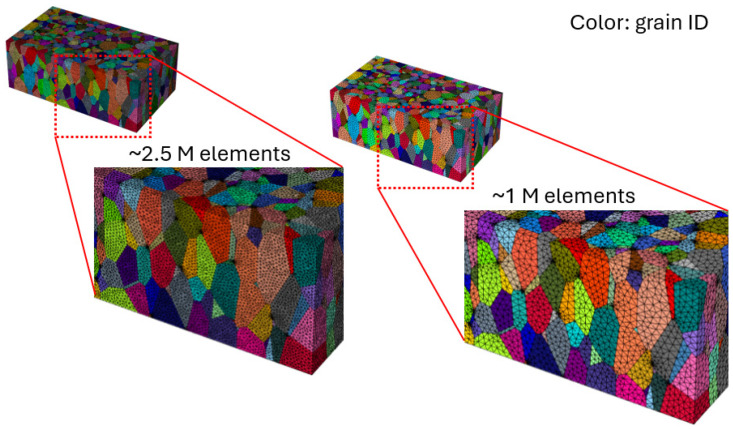
Illustrative comparison of element number in RVE models employing adaptive unstructured tetrahedral finite elements. Colors represent distinct grain identification (ID).

Computational homogenization was employed to determine the mechanical response of an RVE to capture sufficient information about the microstructure and to identify the key factors of the overall material behavior. This approach is particularly useful to derive macroscale material properties from the detailed behavior at the mesoscale [42,43]. The influence of grain morphology on the macroscopic properties is accounted for by solving the equilibrium equations for the entire RVE using the FE method. All RVE models are meshed using unstructured second-order tetrahedral finite elements to achieve a close match to the target sizes and shapes that are as equilateral as possible while maintaining high quality. To accommodate computational disk space limitations, the number of elements is restricted to a maximum of 1.5 million. The primary mesh criterion in this study is that each grain edge must include at least five elements. This is effectively achieved in NEPER by using adaptive mesh functions, which automatically adjust element sizes based on grain edge dimensions, as seen in Figure 10. When representing real microstructures, the RVE often includes numerous small grains that dictate the meshing criteria, with all other grains conforming to relative element sizes. In most cases, this results in fine mesh elements, providing stability during computational virtual testing.

Virtual testing was conducted to determine the macroscopic elastic stiffness which characterizes the relationship between the homogenized macroscopic stress and strain. For all RVE model cases, the displacement load case parameter is set to 10^−3^ to calculate the displacement history.

The macroscopic elastic relationship, expressed in Voigt notation, is given by the following matrix Equation:

(1) $$\left[\begin{array}{c} {\bar{\sigma }}_{11}\\ {\bar{\sigma }}_{22}\\ {\bar{\sigma }}_{33}\\ {\bar{\sigma }}_{12}\\ {\bar{\sigma }}_{13}\\ {\bar{\sigma }}_{23} \end{array}\right]=\left[\begin{array}{c} \;{\bar{E}}_{1111}\\ \;{\bar{E}}_{2211}\\ \;{\bar{E}}_{3311}\\ \;{\bar{E}}_{1211}\\ \;{\bar{E}}_{2311}\\ {\bar{E}}_{1311} \end{array}\begin{array}{c} \;{\bar{E}}_{1122}\\ \;{\bar{E}}_{2222}\\ \;{\bar{E}}_{3322}\\ \;{\bar{E}}_{1222}\\ \;{\bar{E}}_{2322}\\ \;{\bar{E}}_{1322} \end{array}\begin{array}{c} \;{\bar{E}}_{1133}\\ \;{\bar{E}}_{2233}\\ \;{\bar{E}}_{3333}\\ \;{\bar{E}}_{1233}\\ \;{\bar{E}}_{2333}\\ \;{\bar{E}}_{1333} \end{array}\begin{array}{c} {\bar{E}}_{1112}\\ {\bar{E}}_{2212}\\ {\bar{E}}_{3312}\\ \;{\bar{E}}_{1212}\\ {\bar{E}}_{2312}\\ {\bar{E}}_{1312} \end{array}\begin{array}{c} \;{\bar{E}}_{1123}\\ \;{\bar{E}}_{2223}\\ \;{\bar{E}}_{3323}\\ \;{\bar{E}}_{1223}\\ \;{\bar{E}}_{2323}\\ \;{\bar{E}}_{1323} \end{array}\begin{array}{c} {\bar{E}}_{1131}\\ {\bar{E}}_{2213}\\ {\bar{E}}_{3313}\\ \;{\bar{E}}_{1213}\\ {\bar{E}}_{2313}\\ \;{\bar{E}}_{1313} \end{array}\right]\left[\begin{array}{c} {\bar{\varepsilon }}_{11}\\ {\bar{\varepsilon }}_{22}\\ {\bar{\varepsilon }}_{33}\\ {2\bar{\varepsilon }}_{12}\\ 2{\bar{\varepsilon }}_{13}\\ 2{\bar{\varepsilon }}_{23} \end{array}\right]$$

In virtual testing, each component of the macroscopic strain was applied individually, and the resulting homogenized stress provided a corresponding column in the stiffness matrix. This was achieved by prescribing the displacement vector **u** on the boundary Γ of the volume element V, as follows:

(2) $$u=\bar{H}x$$

where $\bar{H}$ is the macroscopic displacement gradient. The strain components are derived from $\bar{H}$ as follows:

(3) $${\bar{\varepsilon }}_{11}={\bar{H}}_{11},{\bar{\varepsilon }}_{22}={\bar{H}}_{22},{\bar{\varepsilon }}_{33}={\bar{H}}_{33},{2\bar{\varepsilon }}_{12}={\bar{H}}_{12}+{\bar{H}}_{21},2{\bar{\varepsilon }}_{23}={\bar{H}}_{23}+{\bar{H}}_{32},2{\bar{\varepsilon }}_{13}={\bar{H}}_{13}+{\bar{H}}_{31}$$

These expressions are based on the following relationship:

(4) $$\bar{\varepsilon }=(\bar{H}+{\bar{H}}^{T})/2$$

**Figure 11:**
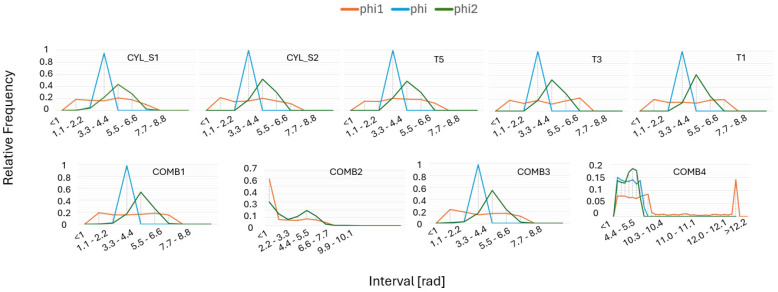
Orientation distributions are utilized as inputs for virtual testing. Further information can be found in Appendix A, which includes visualizations of complete rotational orientation maps implemented in this study.

Prescribing the displacement linearly as $u=\bar{H}x$ on Γ is a convenient option when using the FE method. However, alternative approaches are available in the literature, such as prescribing traction (Neumann boundary conditions) or applying periodic boundary conditions [44]. The corresponding virtual tests, involving the application of linear displacements in both normal and shear directions, are carried out using the RVE models described previously and implementing mean grain orientation distributions as input. In this study, these grain orientations are represented using Euler angles in the Bunge convention [45]. As the standard range is commonly between 0 and 2π, but, as seen in Figure 11, some angles exceed 2π in the MTEX generated grain orientations. This occurs since 3D rotations can be represented by different combinations of angles, allowing values greater than 2π to represent the same orientation. Additionally, MTEX does not automatically reduce these angles to the standard range, leading to values exceeding 2π. In this study, the original values are retained; however, if corrected, they can be adjusted to the standard range by subtracting 2π from the larger angles.

By applying three Euler–Bunge angles, the sample frame becomes aligned with the crystal frame. The first rotation is about the Z-axis (phi1), the second is around the new X-axis (phi), and the third is about the new Z-axis (phi2). This process is repeated for each grain, with all grains sharing the same material properties but exhibiting different grain orientations, collectively forming an RVE. This RVE is then used to calculate the overall homogenized material properties, where homogenized stress $\bar{\sigma }$ is computed from the FE analysis as follows:

(5) $$\bar{\sigma }=\frac{1}{V}\sum_{e=1}^{n_{elem}}V_{e}\sigma_{e}$$

where $V_{e}$ is the element volume, and $\sigma_{e}$ is the average stress in the element.

Three-dimensional CEFE simulations were carried out using ABAQUS 2021 through a user material subroutine (UMAT) [46], and the RVE models were meshed in NEPER (Gmsh) [47] with second-order tetrahedral elements. A cubic symmetric linear elastic material model, as described in the literature [48], was implemented. Consequently, using the Voigt representation, the relationship between stress, strain, and the stiffness tensor can, in the crystal frame, be expressed in matrix form as follows:

(6) $$\left[\begin{array}{c} \sigma_{11}\\ \sigma_{22}\\ \sigma_{33}\\ \tau_{12}\\ \tau_{13}\\ \tau_{23} \end{array}\right]=\left[\begin{array}{c} \;C_{11}\\ \;C_{12}\\ \;C_{13}\\ \;0\\ \;0\\ \;0 \end{array}\begin{array}{c} \;C_{12}\\ \;C_{22}\\ \;C_{23}\\ \;0\\ \;0\\ \;0 \end{array}\begin{array}{c} \;C_{13}\\ \;C_{23}\\ \;C_{33}\\ \;0\\ \;0\\ \;0 \end{array}\begin{array}{c} 0\\ 0\\ 0\\ \;C_{44}\\ 0\\ 0 \end{array}\begin{array}{c} 0\\ 0\\ 0\\ 0\\ \;C_{55}\\ 0 \end{array}\begin{array}{c} 0\\ 0\\ 0\\ 0\\ 0\\ \;C_{66} \end{array}\right]\left[\begin{array}{c} \varepsilon_{11}\\ \varepsilon_{22}\\ \varepsilon_{33}\\ \gamma_{12}\\ \gamma_{13}\\ \gamma_{23} \end{array}\right]$$

where $C_{11}$, $C_{22}$, $C_{33}$, $C_{12}$, $C_{13}$, $C_{23}$, $C_{44}$, $C_{55}$, $C_{66}$ are the 9 elastic constants; $\sigma_{ij},\tau_{ij},\varepsilon_{ij},\gamma_{ij}$ describe the normal stresses, shear stresses, the corresponding normal, and shear strains, respectively. In this study, a simplified model is employed, where, for isotropy, only two independent constants are used, resulting in the following: $C_{11}$ = $C_{22}$ = $C_{33}$, $C_{12}$ = $C_{13}$ = $C_{23}$, $C_{44}$ = $C_{55}$ = $C_{66}$, and for transverse isotropy, five unique constants are required, resulting in the following: $C_{11}$ = $C_{22}$, $C_{33}$, $C_{12}$, $C_{13}$ = $C_{23}$, and $C_{44}$ = $C_{55}$, with $C_{66}$ as additional independent constant.

## 3. Results

### 3.1. Microstructure Representativeness

**Figure 12:**
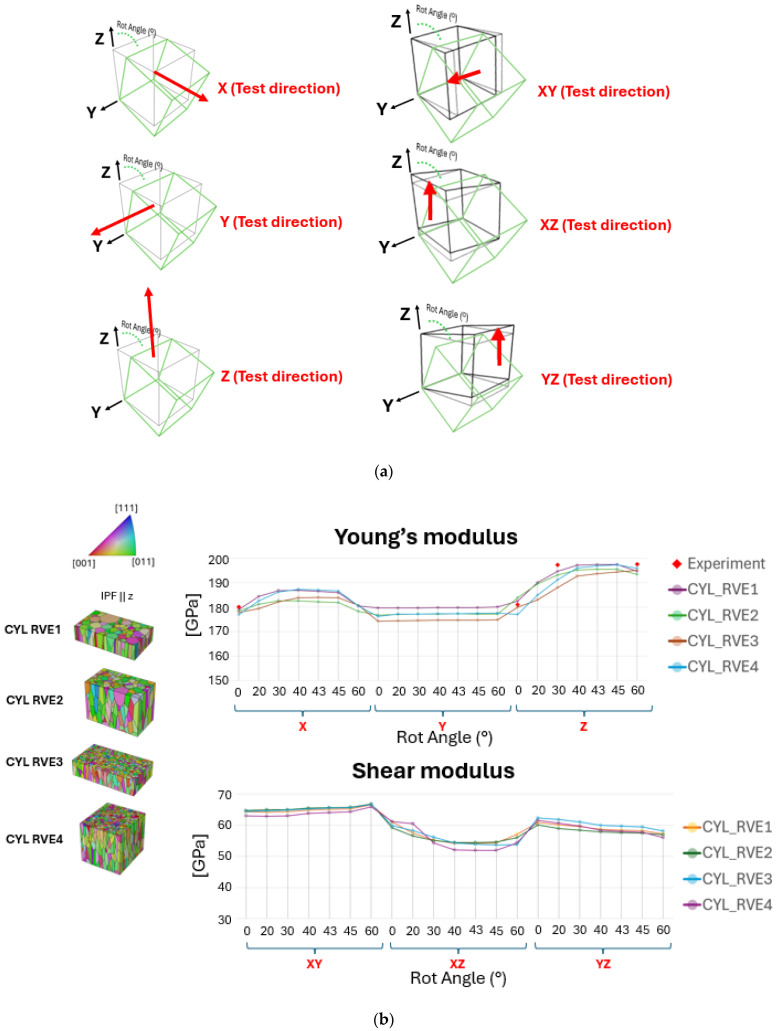

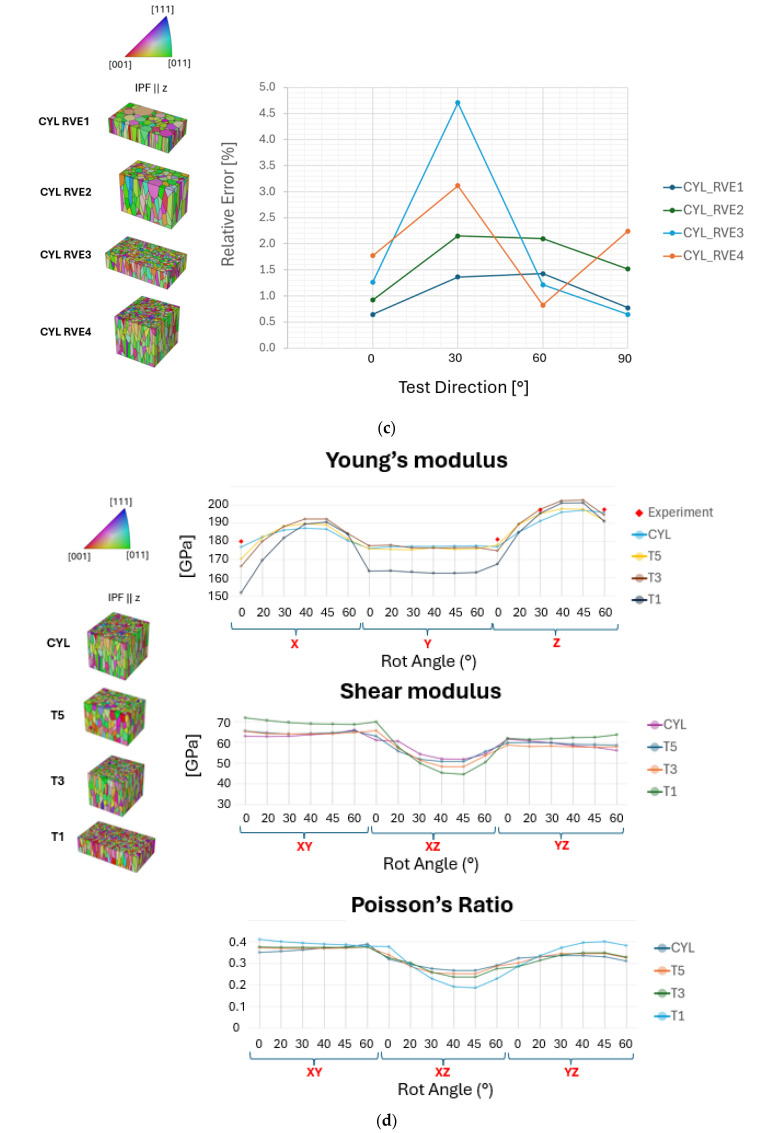

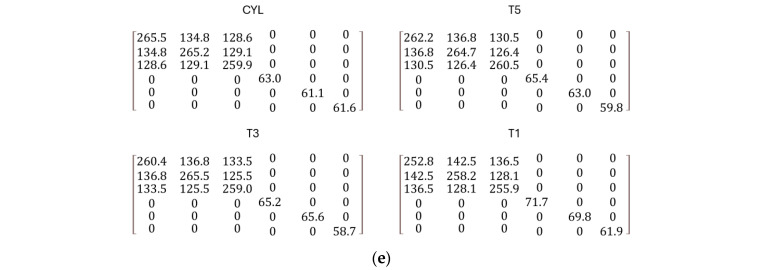
A comparison of the directional properties of (**a**) the various test directions (visualized with red arrows) validating (**b**) the RVE tessellation methods and (**c**) the resulting correlation error, (**d**) sample virtual tests, and their resulting (**e**) nominal stiffness matrices.

Initially, four methods of grain tessellation were evaluated to determine the most optimal approach for further implementation in additional samples. Figure 12a illustrates the different test directions using Equation (2), with components in their original state implemented when rotated at various angles. These angles were analyzed through virtual testing to determine the highest Young’s modulus when rotated around the Y-axis, a scenario not explored in the experimental tests. Figure 12b,d present a comparison of property predictions for the four samples CYL, T5, T3, and T1. The correlation error in the RVE method comparison can be seen in Figure 12c, and the resulting stiffness matrices of virtually tested samples are shown in Figure 12e. The CYL sample, which corresponds to the experimentally tested bulk material, is validated against the resulting experimental measurements of the samples shown in Figure 1a with the following Young’s modulus values: 0° = 180 (GPa), 30° = 197.3 (GPa), 60° = 197.7 (GPa), and 90° = 181 (GPa). The test angles for the samples were selected based on material availability and the feasibility of adhering to specific standard dimensions. However, virtual testing offered a much broader range of possibilities, enabling the material to be tested at a wider variety of angles than feasible with physical samples while still being validated against the available experimental data.

The Young’s modulus values obtained through virtual testing using various representation methods for the CYL sample, as shown in Figure 12b, indicate that grain morphology, such as shapes and sizes, has minimal influence. Instead, grain count and texture are the primary factors contributing to result variations. With an increased grain count, CYL RVE4 approaches the most representative case, CYL RVE1, even though CYL RVE4 does not account for the representativeness of grain size distributions, relying instead on the automatic grain size distribution generated by NEPER.

Figure 12b shows that the RVE1 method, considered the most representative, closely matches the experimental data, suggesting its high potential for accurately predicting material properties. However, despite this strong correlation, the RVE1 method demonstrated significant instability during the tessellation process, making it difficult to apply to other samples. This method requires precise grain morphological inputs, such as grain centroid coordinates, grain sizes, mean grain orientations, and aspect ratios. Given that only a single EBSD plane was considered in this work, it was challenging for NEPER to convert the 2D data into a 3D RVE using this method. As a result, it involved extensive tessellation times and a high risk of mesh generation failures, which limited its practical use. In contrast, the RVE4 method, which involved a broader selection of the EBSD area, showed comparable results to RVE1, with correlation errors of 1.8%, 3.2%, 0.8%, and 2.2% at 0°, 30°, 60°, and 90° test directions, respectively, seen in Figure 12c. Error quantification was calculated using following relative error Equation (notation ‘YM’ corresponds to Young’s Modulus):

(7) $$Relative\;Error=\frac{\left|{YM}_{real}-{YM}_{reconstructed}\right|}{{YM}_{real}}\times 100\%$$

The RVE4 method required only the input of grain orientation and aspect ratios, streamlining the process while achieving a similar level of correlation with the experimental data. By expanding the EBSD area selection, the RVE4 method reduced the complexity and computational demands of the tessellation process, offering a more efficient alternative and thereby used as CYL case in comparison with other samples T5, T3, and T1 shown in Figure 12d. It is also important to emphasize that selecting an EBSD area with a higher grain count was a key factor in improving the accuracy of the property predictions. This approach helped balance the need for a detailed microstructural representation with the practical constraints of the tessellation process, ultimately making the RVE4 method a more feasible choice for virtual testing in other sample cases.

From the results shown in Figure 12d, notably, all cases exhibited similar trends in the Z-direction when the samples were rotated around the Y-axis, revealing consistent anisotropic behavior across all cases. Specifically, the highest values of Young’s modulus were observed at an angle of 40° and 45°, indicating a strong directional dependence of material stiffness. This peak in stiffness at these angles is likely attributed to the alignment of the grain structure along these angular directions relative to the load axis, allowing the material to exhibit maximum resistance to elastic deformation. This kind of directional stiffness variation is characteristic of materials with anisotropic properties which were also evident in these results, as well as shear modulus and Poisson’s ratio, showing corresponding variations at the same angular range. The material response to tension in any given direction may be complex enough to involve two distinct Poisson’s ratios due to the directional dependence of its properties. These findings suggest that the mechanical properties are significantly influenced by the sample orientation, highlighting the importance of considering directional factors when evaluating material behavior. Furthermore, the samples studied demonstrated changes in crystallographic texture as the sample thickness decreased. Specifically, there was an increase in the presence of the <001> crystallographic orientation and a corresponding decrease in the <111> orientation when it was observed perpendicular to the BD. This effect was most pronounced in the T1 sample, which was the thinnest among all the samples. The results suggest that as the sample becomes thinner, there is a shift towards a dominant <001> orientation in this type of microstructure, indicating that the crystallographic texture is strongly influenced by the sample thickness. These shifts in texture are particularly critical in understanding material behavior in applications where directional stresses are significant. Due to these potential effects, further investigation is reasonable, especially in scenarios where non-homogeneity within the material is considered. Investigating combined cases with varying grain textures will help clarify how these factors influence the stress response under different loading conditions. This approach provides a deeper understanding of material performance tendencies across various scenarios, offering valuable insights into material behavior.

**Figure 13:**
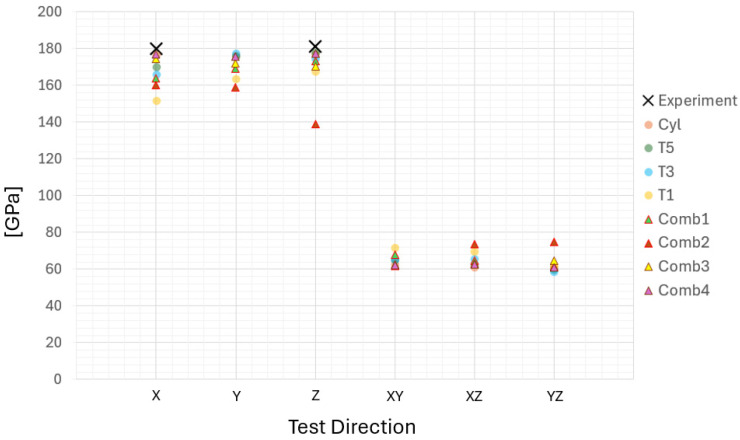
Comparison of directional property results of all studied cases and six tested directions. X, Y, Z correspond to Young’s modulus, and XY, XZ, YZ correspond to shear modulus.

As shown in Figure 13, variations in texture and the combination of different textures lead to significantly different outcomes for altered test directions.

The COMB1 case, which combines the CYL and T1 samples, produces results that are intermediate between the two, as expected. However, when combining the CYL texture with the ideal <001> texture, there is a notable decrease in stiffness in the Z-direction, while the stiffness in the X- and Y-directions remains comparable to the levels observed in the T1 sample. In the COMB2 case, the highest anisotropy is observed, with the lowest stiffness in the Z-direction and the highest stiffness in the XZ- and YZ-directions compared to all other cases. The COMB3 case, which combines the CYL sample with material data from a previous PBF-EB study [13], resulted in a weak anisotropic behavior. However, it was closer to the isotropic behavior observed in the CYL texture. COMB4 case, which integrates segments of randomly oriented grain structures with strongly textured <001> orientations, exhibited a clear isotropic behavior. This combination resulted in a material that demonstrates uniform properties in all directions, contrasting with the directional dependencies observed in other textures. The random orientation of grains alongside the distinct <001> texture effectively balanced out anisotropic effects, leading to consistency regardless of the test direction.

### 3.2. Stress and Strain Distributions

**Figure 14:**
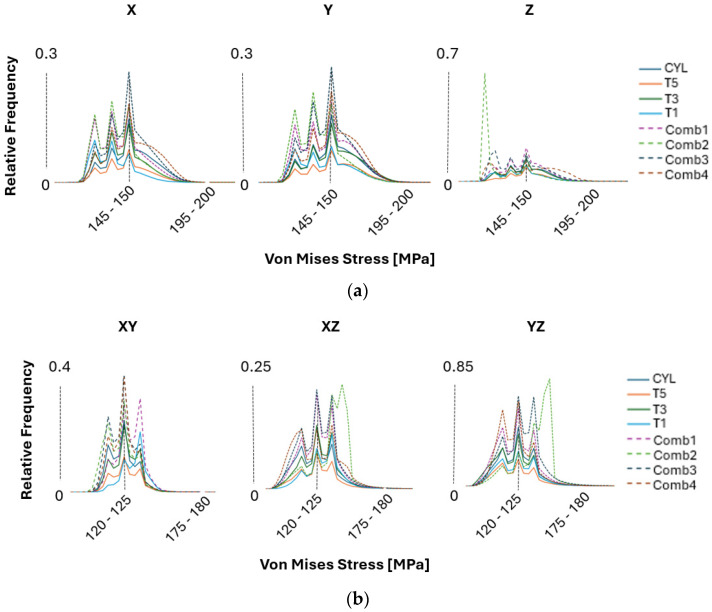
Comparison of von Mises stress distributions across all sample cases studied when tested in (**a**) normal and (**b**) shear directions.

The stress and strain distributions were analyzed by comparing all samples using RVE models and examining the 2D sections of the previously described combined cases. Figure 14 illustrates the von Mises stress distributions and the relative frequency of stress intervals across these samples. A higher frequency and narrower stress distribution indicates a stronger texture effect, leading to more uniform material behavior. This suggests that the texture and orientation of the grains significantly influence how stress is distributed throughout the material, with increased texture leading to more stable and predictable stress distributions. However, this tendency is most pronounced in the Z-direction, where the grain orientation is predominantly <001> and parallel to the BD.

Observations of stress distribution results reveal that introducing increased texture within the grain structures leads to a more uniform material behavior. When the texture is less pronounced, the relative frequency of stress levels decreases, indicating a more variable stress interval. The samples with less texture, such as in the CYL sample, exhibit a higher degree of non-uniform stress distribution, as indicated by the increased variability in stress levels. In contrast, samples with strong texture, such as in the COMB2 case, show elevated frequency levels, corresponding to a more consistent stress distribution. This suggests that a more pronounced texture within the grain structure contributes to a more homogeneous distribution of stress across the material. However, the analysis of combined structures focuses only on the overall material behavior and does not evaluate localized stress concentrations at the interfaces, which could be crucial in cases where crack propagation or similar phenomena are of interest.

The multimodal behavior observed in the stress distributions arises from the combined presence of <001>, <011>, and <111> crystallographic orientations normal to the planes being studied. This combination of orientations leads to a complex, layered interaction within the material, which ultimately contributes to a more non-uniform distribution of stress across different directions. As a result, all PBF-LB samples exhibit this characteristic, resulting in isotropic material behavior where this isotropy is directly influenced by the balanced mix of crystallographic orientations, which mitigates any directional dependence in the material response to stress.

**Figure 15:**
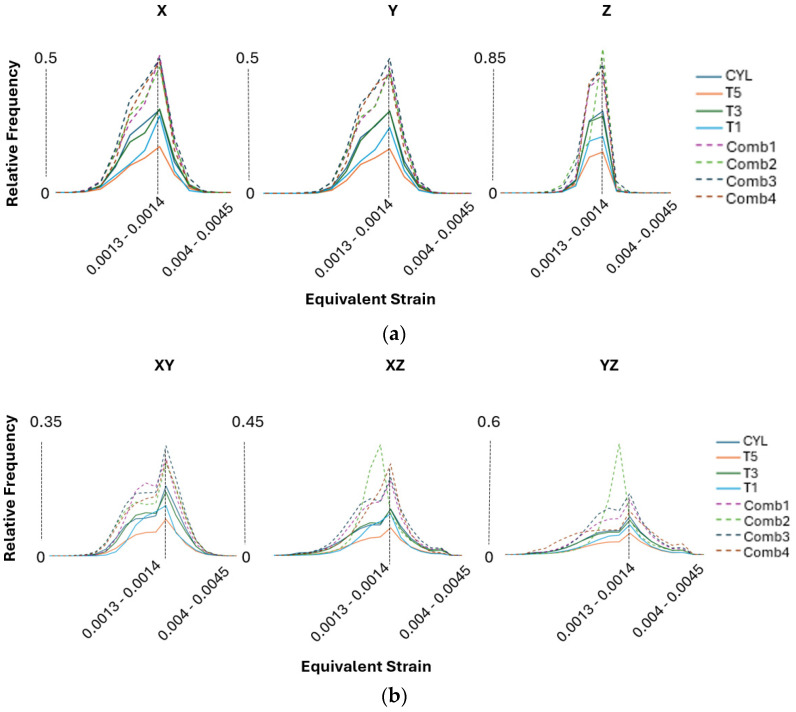
Comparison of equivalent strain distributions across all sample cases studied when tested in (**a**) normal and (**b**) shear directions.

The strain distribution results shown in Figure 15 mirror the patterns observed in the stress distribution analysis, particularly concerning the relative frequency levels. As the frequency of strain points increases, it indicates a more uniform strain distribution throughout the material. This suggests that, similar to the stress distribution, a higher frequency corresponds to more consistent deformation behavior across different regions of the sample. The uniformity in strain distribution highlights the predictable response to external forces, reinforcing the conclusion that certain textural characteristics contribute significantly to a more homogeneous mechanical performance when considering the global macroscale level. More interestingly, the material exhibits bimodal characteristics, particularly when tested in shear directions, as shown in Figure 15b. This bimodal behavior suggests that the material response to shear stress is influenced by the presence of two distinct peaks in the strain distribution. This could be due to varying grain orientations or differences in local texture that cause the material to react differently under shear loading.

The presence of strain peaks indicates that different regions within the material may have distinct mechanical responses, which can result from the complex interplay of crystallographic orientations, grain boundary interactions, or localized stress concentrations under shear conditions. This bimodal pattern indicates that the material does not deform uniformly under shear stress but instead exhibits two separate modes of response. These modes could correspond to different sets of grains or microstructural features that align differently relative to the applied shear forces. For example, grains with orientations that are more resistant to shear may form one peak, while those that are less resistant form another, resulting in a distribution with two distinct maxima. The presence of bimodal characteristics in the shear directions also implies that the material performance could vary significantly depending on the specific loading conditions. In practical applications, such behavior could influence the material toughness, fracture resistance, or fatigue life, depending on how the shear forces interact with the underlying microstructure. This highlights the importance of understanding the microstructural texture and grain structure to predict its behavior more accurately under different mechanical loads. It is important to note that in the PBF-EB study [13], the bimodal behavior in strain distributions was less pronounced compared to the PBF-LB samples. This suggests that the material produced by PBF-EB is less affected in the shear directions and exhibits greater resistance to potential fracture, indicating a more stable structure under shear loading conditions.

### 3.3. Non-Homogeneous Structure

**Figure 16:**
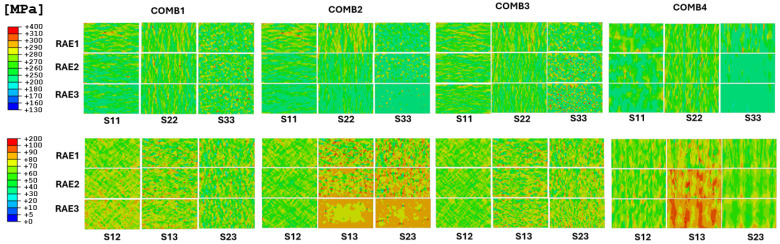
Comparison of representative area element (RAE) sections in stress distributions of combined cases in all test directions.

Non-homogeneous structures, referred to as combined structures, are evaluated using the validated methodology (M3) outlined in the preceding sections. For the assessment, all cases involving combined structures are compared by selecting RAE sections at distinct positions along the Z-axis, as illustrated in Figure 16. This approach allows for a detailed analysis of the equivalent stresses by plotting them to visualize the concentrated locations within the material. The analysis further demonstrates how these concentration points vary under different scenarios and how they are distributed globally within the corresponding RVE.

The stress result analysis for S11, S22, S33, S12, S13, and S23 in the X, Y, Z, XY, XZ, and YZ test directions, respectively, illustrates the stress level distributions at each node within the element. These results are gathered from the direction in which the test was conducted, as it is the most impacted direction when comparing all results and therefore most relevant for evaluation. Analysis of the high-stress distributions in COMB1 indicates that the RAE1 (CYL) of the interface region in S11, S22, and S33 experiences the most significant stress concentration. The stress distribution is similar across all directions due to the isotropic nature of the material. In contrast, the stress distributions in the same directions for the T1 textured part (RAE3) show differing effects, with S11 exhibiting a distinct stress behavior compared to S22 and S33, due to the anisotropic nature of this structure. However, shear stress responses in S12 of RAE3 show increased levels compared to the RAE1 part.

When analyzing the stresses in the COMB2 structure, S11 and S22, particularly at the interface (RAE2), experience less impact in these directions compared to other cases. However, RAE1 in those directions develop the highest stress distribution and RAE3—lowest stress distribution compared to all other cases. It is observed that the COMB2 structure type increases the risk of localized stress concentration in the CYL part (RAE1) compared to the CYL part in COMB1 structure type. A similar pattern in S11 and S22 is observed in the COMB3 case. However, in this specific structure type, the stress concentration in S33 increases as the texture of the <001> crystallographic orientation (RAE3) becomes more pronounced, resulting in the worst outcome of this specific direction compared to other cases.

In the COMB2 case, the shear stress distributions in S13 and S23 increase in the RAE1 and RAE2 planes while showing a consistently uniform stress response in the RAE3 plane due to the influence of the texture. The shear stress distributions in the COMB3 case exhibit similar tendencies across all shear directions, regardless of the texture transition in this specific structure. This combination of PBF-LB-processed material with PBF-EB-processed material in the COMB3 configuration emerges as a surprisingly effective option when considering stress distributions in shear directions, particularly in comparison to the COMB1 case where the PBF-LB-processed material of two distinct textures is combined.

In the COMB4 case, as shown in Figure 16, high-stress distributions are observed in the S11, S22, and S33 directions at the interface (RAE1). Notably, the S22 results exhibit similar outcomes regardless of the interface position, while highly affected stress regions develop in the S13 shear direction. The high-stress distributions in this case are primarily due to the presence of varying textures and multiple interfaces within the same RVE. These varying textures result in both anisotropic and isotropic material behavior simultaneously, with different grain orientations reacting differently to applied stresses. The presence of multiple interfaces amplifies this effect by introducing discontinuities and misalignments between grain structures, leading to localized stress concentrations. This interplay of diverse grain textures and interfaces causes unpredictable material responses, forming distinct stress and strain patterns and making this case the worst-performing in terms of mechanical behavior among the cases studied in this work.

This contrast underscores the variability in stress distribution across different directions and highlights the challenges associated with the AM process control. This variation is likely attributed to the transitional nature of grain orientations and textures in this region, leading to anisotropic behavior across different directions. In certain directions, the interface region may contain grains more aligned with the applied stress, resulting in higher stress concentrations. Conversely, other areas of the interface, with differing grain orientations or boundary configurations, may exhibit a different stress response, particularly due to grain misalignment or weaker grain boundaries

**Figure 17:**
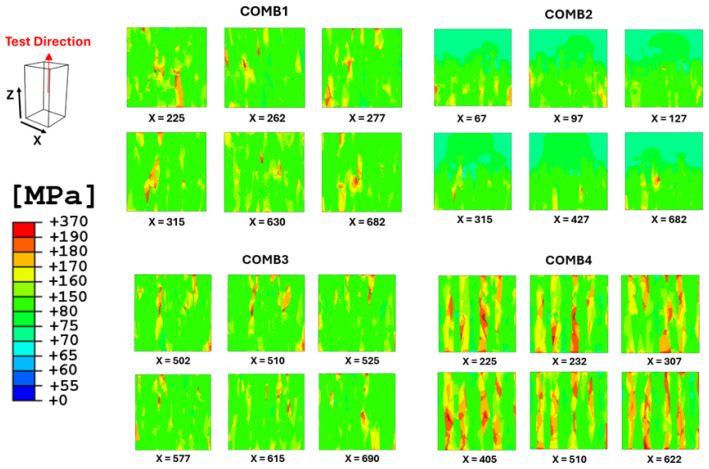
Comparison of von Mises stress distribution results of most affected areas normal to X-axis in combined cases.

Von Mises stress levels were also analyzed for all combined cases, as shown in Figure 17, offering additional insights into global material behavior and the interaction between different structural configurations.

These results are derived from the most affected planes in normal to the X-axis, focusing on the material subjected to loading in the Z-direction. This approach offers valuable localized insights into stress behavior at the interface between the two distinct structures in the combined cases studied. As observed, this behavior varies across different configurations. In the COMB1 case, high-level stresses occur at the interface area, with stresses mitigating in the middle part of the RVE. In the COMB2 case, stresses also begin at the interface but then mitigate toward the lower part of the RVE, which features the CYL structure. In the COMB3 case, where PBF-LB is combined with PBF-EB material, the stresses primarily mitigate in the upper part of the RVE, which corresponds to the PBF-EB-processed material. Lastly, in the COMB4 case, stresses seem to be distributed at every interface of each segment, highlighting significant issues at the interfaces within this structure. These differences in high-level stress distribution among the cases can be attributed to the unique microstructural characteristics and grain orientations present in each configuration. The von Mises stresses in the middle part of the COMB1 RVE suggests that the interface area efficiently channels stresses, allowing for a smooth transition. The microstructure here likely has more favorable grain orientations that align well with the applied load, promoting effective load transfer. In the COMB2 case, the shift of stress mitigation to the lower part of the RVE indicates that the CYL crystallographic texture is better at absorbing and redistributing stresses. The grain orientations in this region may facilitate stress relaxation, which contrasts with the interface area where stresses initially concentrate. The case COMB3 of PBF-LB and PBF-EB combination results in stress distribution in the upper part of the RVE. This implies that the PBF-EB-processed material possesses characteristics that are able to accommodate stresses more effectively than the PBF-LB structure, possibly due to its enhanced strength and toughness. The initiation of stresses at every interface of COMB4 case signifies a lack of strength and stability within the microstructure. The presence of multiple interfaces and varying textures creates discontinuities that intensify stress concentrations, making this configuration more prone to localized failures.

## 4. Discussion

Initially, this work explores various methods for representing grain structures of PBF-processed material, which is the continuation of the previous study referenced in [13]. The objective is to develop a methodology that accurately replicates actual grain morphology while considering the capabilities of NEPER for grain tessellation and RVE model creation. By comparing four RVE tessellation methods using a single EBSD data plane normal to BD, we aim to identify the most effective method for representing the Hastelloy X material grain structures. The chosen most optimal method is then applied across all samples, ensuring consistency and stability during the tessellation process. Additionally, the combined structures are artificially generated in a specific way integrating the real EBSD data. Alternative 3D microstructure representation methods, such as the two-point correlation function, also referred to as pair correlations in scattering theory, have been studied by Tabei et al. [16], showed a close correlation with the experimental data. Although this method uses statistical functions to compare the reconstructed sample to the real material incorporating only four 2D microstructure planes, it would not be suitable for representing local microstructural changes that introduce non-homogeneity, such as anomalies or texture-breaking features, without the use of advanced equipment like high-energy X-ray diffraction microscopy [49]. Our study explores different approaches for modeling complex grain structures, with the primary aim of accurately capturing realistic 3D grain morphology using a single 2D EBSD dataset and the tessellation capabilities of NEPER while also examining the non-homogeneity commonly found in AM materials. Hastelloy X material and PBF-LB process were selected due to the resulting complex microstructure where grain morphology and texture makes it suitable for evaluating the presented methods. This selection also aligns with industry needs, where accurate 3D grain representation can directly assist in analyzing the performance of produced materials. Industries such as aerospace and energy, which demand high-performance applications, would benefit from this research, particularly in predicting material properties and estimating performance before the actual material build. The focus on Hastelloy X in this study explores tessellation methods addressing different challenges in replicating grain orientation, size, and morphology accurately within 3D RVE models. The iterative optimization-based approach in NEPER allows for a precise 3D representation that closely mimics the EBSD data. However, the computational limitations and required precision are significant barriers to directly implementing the most representative microstructure. Among the evaluated methods, M1 was the most representative in terms of matching experimental data. However, this method approach involved considerable instability during tessellation, largely due to its need for precise grain data inputs such as centroid coordinates, grain sizes, orientations, and aspect ratios. This dependence on detailed grain morphology, combined with the challenge of converting 2D EBSD data into a 3D RVE model, leads to a time-consuming tessellation process and is prone to meshing failures. As such, its practicality was limited despite its accuracy. Methods like M1 aim for high accuracy but encounter limitations in optimization due to the extensive input data. Simplified methods streamline tessellation but may lose some fidelity in grain positioning and morphology. However, it has been shown that the coarser representation using M3 produces results consistent with those obtained from the M1 tessellation method. This study demonstrates that texture information and grain count within the RVE are key factors influencing representativeness in the bulk material. The balance between microstructural accuracy and tessellation efficiency made M3 a feasible option for further virtual testing, particularly in scenarios requiring a larger EBSD area and a higher grain count, which a previous study [13] identified as a limitation when handling extensive EBSD data.

A key research gap addressed in this study is the lack of clear methodologies for generating 3D non-homogeneous grain structures from minimal input data, which limits the ability to perform large-scale and cost-effective studies. Previous studies utilizing techniques like EBSD serial sectioning [39] have demonstrated that reconstructed sections can closely match directly measured EBSD data in terms of microstructural parameters such as grain size, morphology, image quality, and kernel average misorientation distribution. Another study [50] outlines the complete process of simulation, starting from microstructure extraction using 3D tomography and property determination of individual phases via nanoindentation to the development of a simulation model and its validation through experimental data. However, these approaches are time-consuming, require costly equipment, and demand a high level of expertise, making them challenging to implement effectively in industrial environments.

A study by Randle and Engler’s [51] provides a comprehensive summary of several investigations, emphasizing the representativeness of EBSD data based on the number of single-grain orientations analyzed. These studies highlight that critical microstructural features, such as texture, depend on the number of grains captured in EBSD measurements. Studies on weaker textures suggest that approximately a thousand grains may be needed for reliable characterization [52]. Similarly, Davut and Zaefferer [53] investigated the representativeness of EBSD data in determining phase fractions in transformation-induced plasticity (TRIP) steels, underscoring the importance of sufficient grain sampling. To balance numerical efficiency with accuracy, methods to optimize RVE size while preserving its representativeness have been proposed by Nakamachi et al. [14] and Pélissou et al. [54]. It is widely recognized that, in any statistical analysis, a sufficiently large sample size is necessary to minimize artifacts and biases. This principle also applies to experimental data used in micromechanical modeling, ensuring reliable predictions. In the study by Biswas et al. [52], conduct a comprehensive study to evaluate the impact of EBSD data on the outcomes of a micromechanical model within the crystal plasticity finite element (CPFE) method framework. A key focus is determining the adequate number of grains required in EBSD measurements to achieve representative results in micromechanical simulations. To this end, various EBSD scan sizes were performed on AM 316L stainless steel. Microstructural features, such as texture and grain size, were extracted from the EBSD dataset to create a synthetic microstructure to numerically predict the material mechanical behavior.

In our study, we have also shown that a single-section approach could simplify model setup while still accurately representing essential grain characteristics. Additionally, this study aims to clarify the advantages and limitations of each tessellation method, ultimately highlighting the effectiveness of a combined RVE approach for Hastelloy X. This approach incorporates various grain texture configurations, a topic that has not yet been explored. Our study therefore provides insight into how different RVEs and tessellation methods contribute to material modeling accuracy, focusing on how the simplified NEPER models and selective grain orientation data can enhance non-homogeneous 3D microstructural representation. In this study, different samples provide a basis for understanding how grain orientations evolve, particularly as the structure changes in the sample thickness. In the CYL sample, the grain orientation closely mirrors that of the bulk material sample through experimental validation, where it resulted in <001> aligned parallel to the X-direction, <111> along the Y-direction, and <011> in the Z-direction. This pattern results in a material with isotropic characteristics but still retains some directional texture in the BD. However, as the structure becomes thinner, more pronounced texture changes emerge, leading to slight anisotropy where the <001> orientation starts dominating, especially along the X- and Y-planes. The texture changes observed emphasize the significant role that thickness plays in microstructure evolution. This shift in texture indicates how grain orientation can be influenced by not only material dimensions but also process parameters shown in the PBF-EB study [13] and highlights the need to study the interaction of different textures within the same AM process.

Understanding these variations allows for optimized process control, enabling tailored material performance based on specific application needs. As different textures interact, this study investigates how these interactions affect material behavior and properties at the macroscale. To do this, the artificially generated combined microstructures, representing transition regions between various grain structures, are evaluated. Although these microstructures are artificially generated, the input grain data are primarily based on real EBSD data, except for the special case of COMB4, which represents a synthetic combined-grain structure. These combined structures are then analyzed by extracting information from 2D RAE sections at various positions within the 3D RVEs. The gradual transition regions of these grain structures create an important area of study for applications requiring customized material properties.

The analysis of the virtual test results from all generated RVE cases provided significant and valuable insights into the material behavior and properties. In the case of the CYL sample, the results showed a consistent correlation between virtual testing and bulk material experimental data. The highest material stiffness was observed at angles of 40° and 45° in all samples when rotated around the Y-axis, suggesting a strong directional dependence of the elastic properties when tested along the Z-direction. This directional dependence is key to understanding how different grain orientations and textures influence mechanical behavior. Further investigation into the combined microstructures, characterized by non-homogeneity, revealed that, for example, combining the CYL and T1 textures in the COMB1 case resulted in predicted elastic properties that reflected the characteristics of both textures. Conversely, the COMB4 case, characterized by a balance between random and strongly <001> textured grains, exhibited isotropic global behavior. This shows how the specific combination of textures and orientations can either enhance or reduce anisotropic properties. This study also explored stress and strain distributions to further understand the role of texture in material behavior. The results highlight that a more pronounced grain texture leads to a more uniform stress distribution, particularly in the Z-direction, where the <001> orientation dominates. In contrast, materials with less texture showed greater variability in stress and strain distributions, indicating non-uniform material behavior. This variability is particularly noticeable in the PBF-LB samples, where bimodal characteristics in strain distributions under shear loading suggest distinct mechanical responses in certain regions of the material. Such behavior, driven by variations in grain orientation and local texture, underscores the need for further research into how shear forces interact with the microstructure, especially in applications that require high toughness and fracture resistance. Stress analysis in the COMB1 structure revealed that the CYL part (RAE1) exhibited the highest stress concentrations across S11, S22, and S33 directions, attributed to its isotropic nature. In contrast, the T1-textured part (RAE3) showed distinct stress behaviors, especially in the S11 direction, which indicates an anisotropic response. In the COMB2 case, the CYL part (RAE1) exhibited the highest stress distribution, emphasizing the importance of structural design to mitigate stress concentrations. These results highlight the role of material texture in determining stress distribution and the need for tailored designs to optimize performance. Further analysis of the COMB3 and COMB4 cases provided additional insights into the influence of crystallographic orientation. In COMB3, stress concentrations in S33 were amplified due to the pronounced <001> crystallographic orientation (RAE3). However, in COMB4, the interaction between various textures led to high stress distributions across S22 RAEs, indicating complexity and sensitivity in the transition region due to grain misalignment and boundary discontinuities. When examining high-level stress behavior, distinct patterns emerge. In COMB1, stresses were mitigated in the middle of the RVE section, indicating a smooth transition under loading. In COMB2, stresses at the interface dissipated towards the lower part of the RVE, suggesting effective stress absorption by the CYL-textured structure. In contrast, COMB3 showed a more favorable stress distribution in the upper section, demonstrating the strength of the PBF-EB material. However, COMB4 exhibited high-level stresses at every interface, indicating significant cohesion and stability issues, leading to an increased risk of localized failure.

## 5. Conclusions

This study provides a comprehensive evaluation of different grain tessellation methods, highlighting the trade-offs between accuracy, efficiency, and stability. While methods that incorporate extensive real grain data provide highly accurate representations of the actual microstructure, they are time-consuming and prone to instability. However, they would be necessary for a study focused on representing combined grain interfaces using real microstructures. With the input of multiple EBSD data planes, the tessellation process would be less problematic compared to the current case. In contrast, methods with less actual input data, such as M2 and M3, demonstrated that a more efficient tessellation process is possible while still maintaining reasonable accuracy, as shown in this specific study. The study of transition regions and combined microstructures reveals the complex interaction between different grain orientations and textures, which significantly impacts material behavior. Understanding these transitions is crucial for optimizing material properties in applications requiring specific or tailored grain structures.

The stress and strain analysis results underscore the critical role of microstructural characteristics and grain orientations in determining the mechanical behavior of the various textures. The variability in stress and strain distributions highlights the need for understanding how different materials respond to applied loads, especially in applications where directional performance is crucial. The insights gained from studying the combined structures establish a foundation for future research aiming for a more detailed investigation of the interface region and its representation. This effort seeks to address the challenges posed by non-homogeneous structures and to investigate the prediction of local properties at their interfaces.

## Figures and Tables

**Table 1:** Chemical composition of Hastelloy X.

Ni (wt%)	Cr (wt%)	Fe (wt%)	Mo (wt%)	W (wt%)	Co (wt%)	C (wt%)	Si (wt%)	Mn (wt%)
Balance	20.93	17.89	8.73	0.84	1.46	0.01	0.18	0.01

**Table 2:** Grain data characterization settings used in MTEX.

	CYL	T5	T3	T1
Resolution (pixel)	1148 × 754	285 × 204	765 × 543	1071 × 569
Denoising *	no	yes	yes	no
Threshold Angle (°)	10	5	10	7
Step size (µm)	1	4.4	1.5	0.7
Aspect Ratio (BD)	3.7	2	3.7	3.9

* Denoising applied to repair missing pixel information.

**Table 3:** The RVE model setup used in the studied cases, including the tessellation method and the description of each method described in the following subsections.

	CYL RVE1	CYL RVE2	CYL RVE3	CYL RVE4	T5	T3	T1	Comb1	Comb2	Comb3	Comb4
Tessellation Method	M1 *	M2 **	M3 ***	M3	M3	M3	M3	CM ***	CM	CM	CM
Grain count	1018	1018	1018	1866	1061	1850	1445	2463	2463	3414	2463
RVE thickness (µm)	200	400	200	400	200	400	200	400	400	400	400
Element count	375,418	555,911	292,375	639,414	336,403	609,910	440,530	917,430	917,430	1,145,013	917,430

* Input: centroid coordinates, grain sizes, mean grain orientations, aspect ratio; ** Input: grain sizes, mean grain orientations, aspect ratio; *** Input: mean grain orientations, aspect ratio.

**Figure A1:**
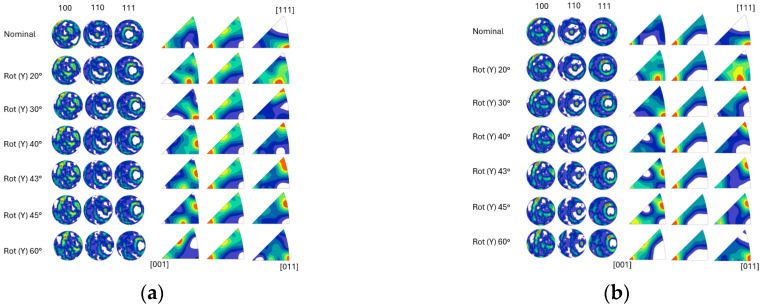

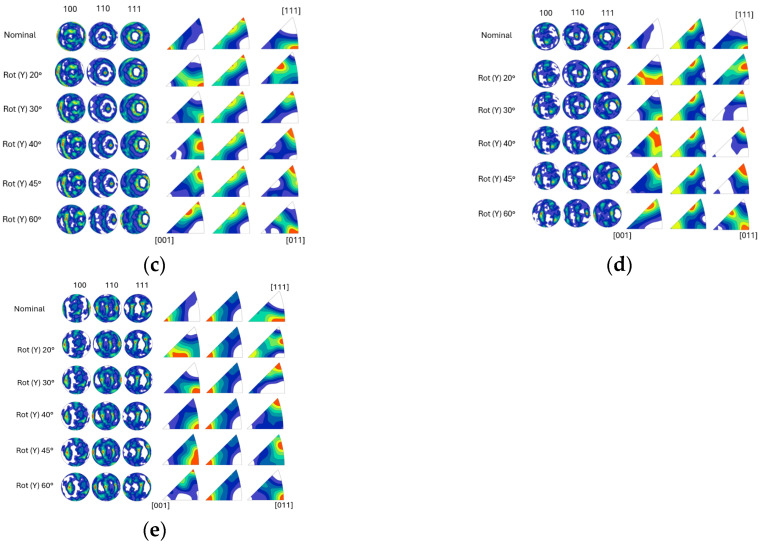
Pole figure (PF) and inverse pole figure (IPF) maps showing the grain orientation of all rotations around Y-axis used as input for (**a**) CYL sample EBSD S1, (**b**) CYL sample EBSD S2, (**c**) T5 sample, (**d**) T3 sample, and (**e**) T1 sample. Notation “Nominal” refers to 0° rotation. Color represents the grain orientation crystallographic direction.

## Data Availability

Data are contained within the article. The raw data supporting the conclusions of this article will be made available by the authors on request.
